# The Evolving Role of Triage and Appointment-Based Admission to Improve Service, Care and Outcomes in Animal Shelters

**DOI:** 10.3389/fvets.2022.809340

**Published:** 2022-03-04

**Authors:** Kate F. Hurley

**Affiliations:** Koret Shelter Medicine Program, School of Veterinary Medicine, University of California, Davis, Davis, CA, United States

**Keywords:** animal shelter, managed access, community oriented, coordinated care, appointment, triage system, stray dog and cat, animal relinquishment

## Abstract

The historical norm for many animal shelters has been to admit animals on an unscheduled basis, without prior assessment of animal or client need or regard to the shelter's ability to deliver humane care or ensure appropriate outcomes. This approach allows little opportunity to provide finders or owners with alternatives to keep pets safe in their homes or community rather than being impounded. In addition to needlessly impounding animals and separating pets from families, unmanaged/unscheduled admission frequently results in animal influx exceeding shelter capacity, leading to crowding, stress, disease, and euthanasia of animals, as well as poor customer experience, compromised staffing efficiency and decreased organizational effectiveness. Many of these harmful consequences disproportionately impact vulnerable community members and their pets. Triage and appointment-based services have been well developed in healthcare and other service sectors allowing organizations to prioritize the most urgent cases, align services with organizational resources and provide situation-specific solutions that may include virtual support or referral as appropriate. This article discusses the trend in animal sheltering toward triage and appointment-based services that parallels the use of these practices in human healthcare. Reported positive results of this approach are detailed including improved staff morale, reduced disease rates and substantially reduced euthanasia. These positive outcomes support the endorsement of triage and appointment-based services by multiple North American animal welfare professional and academic organizations, recognizing that it better realizes the goals of shelters to serve the common good of animals and people in the most humane, equitable and effective possible way.

## Introduction

“Triage” is a well-developed strategy in human general practice medicine and refers to the provision of care based on exigency and available resources. Triage allows the medical practice to prioritize those who are most in need of immediate care, and who benefit most from clinic services (1). In order to make this determination, the following factors are considered:

- Why the patient has sought help from their general practitioner
- What kind of help the patient needs (which may be different than the reason help was sought)
- How quickly the patient needs help
- Who the best person is to help this patient

The answers to these 4 questions allow a determination of where and when a patient should be accommodated. Urgent cases can be routed directly to immediate care, non-emergencies can be scheduled for a time when the practice is able to accommodate the patient's needs, and some cases can be identified that can be handled more conveniently and efficiently through remote services, for example through a telehealth visit or call with an advice nurse (2). This in turn has resulted in decreased workload for primary care physicians while increasing speed of access to information for patients (3).

Although it may not always provide instant gratification, the benefits of triage can be readily appreciated by users of health care services. Few general practices—human or veterinary—would be able to safely accommodate all patients seeking care on a walk-in basis. We appreciate the ability to be seen on an appointment basis to avoid long waits, and hope that the health systems we participate in are not so overwhelmed that they are unable to accommodate emergencies. We recognize that remote care can be a safe and convenient option while also lowering costs and risks within healthcare systems.

## Triage in Animal Shelters

In spite of its apparent benefits in other sectors, triage systems have not historically been well developed in North American animal shelters. Instead, the norm has been *ad hoc*, unscheduled admission of any animal presented during open hours (and sometimes even after hours via “drop boxes”) regardless of shelter capacity or animal needs. In fact many publicly funded shelters report being explicitly prohibited from managing the flow of animals into the shelter. Yet animal shelters share the challenges faced by human and veterinary general practice in terms of the number and variety of animals presented for care, from urgent (e.g., injured and dangerous animals) to more chronic (e.g., free roaming neighborhood cats). Without a triage system in place, shelters tend to fill with less critical cases, leaving resources stretched thin when emergencies do occur. Crowding in shelters leads to increased disease and behavioral disorders, compounding the strain on limited resources.

Perhaps most importantly, in the absence of thoughtful triage animals are impounded that could have been served more humanely, effectively and equitably by remaining in the community. For instance, research has documented that most stray dogs are found close to their homes and that dogs and cats are >11 and >40 times, respectively, more likely to be found by searching the neighborhood of origin or returning home on their own than through a call or visit to a shelter (4, 5). By transporting found animals to a shelter without first making efforts to locate the owner, well-intended finders often unwittingly reduce the likelihood of the pet ever being reunited with its family. This is especially likely where language, transportation or other barriers prevent some community members from readily accessing the shelter, perhaps one reason why those making under $30,000 USD annually were less than half as likely to find a lost pet than those making over $50,000 (4).

Shelter admission may likewise not be the best option for pets whose owners are considering relinquishment; for underage kittens found outside; for healthy community cats, or for other common categories of animals presented to shelters. For instance, it may be possible to provide support to keep pets safely with their families or help owners rehome their own animal without the stress and risk of surrendering to a shelter, while underage kittens may be safer remaining with their mother until they are old enough for adoption. Such ideal solutions may not always be possible, but cannot even be considered without a process in place to evaluate the animal and owner or finder's situation and offer alternatives to admission if appropriate.

### Trends Toward Triage

In recent years there has been an increasing appreciation for the value of triage in animal shelters to provide situation-specific solutions. Programs utilizing triage have been known by various terms, including Community-oriented Sheltering, Managed Admissions/Intake, Coordinated Entry and Appointment-based Services. In the last decade such programs have been implemented by a number of shelters and incorporated into national initiatives. Managed Admissions (with provision of Alternatives to Intake) was among the key initiatives of the Million Cat Challenge, a successful campaign to save over a million cats from euthanasia in North American animal shelters over a 5-year period from 2014 to 2019 (6). Managed Admissions was also identified as a component of the Capacity for Care management model, which has been linked to reduced disease, lower costs and reduced euthanasia rates (7).

The operational changes necessitated by the COVID-19 pandemic accelerated the adoption of triage systems by North American animal shelters (8). Many shelters implemented appointment-based services out of necessity to regulate customer flow and keep public and staff safe. An unexpected result was the greater opportunity to provide a thoughtful assessment of animal needs, offer alternatives to impoundment and ensure operation within shelter capacity. The benefits were evident in reduced euthanasia (9, 10) and improved animal care in the shelter, leading to the retention of this practice in some cases even as pandemic restrictions eased. For instance, the Managed Intake program implemented in 2020 by the Los Angeles County Department of Animal Care and Control, one of the largest shelter systems in the U.S., earned awards in 2021 from the National Association of Counties and from the Quality and Productivity Commission, and received the endorsement of national organizations including the American Society for Prevention of Cruelty to Animals, Best Friends Animal Society, and the National Association for Animal Care and Control (11).

### Evolution of the Shelter Triage Model

Building on the success of appointment-based services, the “Human Animal Support Services” (HASS) animal sheltering model emerged in 2020 with a focus on providing safety net services to keep animals in homes, not kennels (12). Key strategies of the HASS model include community-based interventions that bypass shelter intake, such as returning lost pets to their homes in the field, increasing the scope of animals placed in to foster care, and overcoming barriers to keeping pets in their original homes (13). Some shelters have chosen to rebrand themselves as “Pet Resource Centers” to reinforce the idea that impoundment is not a one-size-fits-all solution.

Similar to the human medicine model, triage in a shelter context means that each time an animal is presented for possible intake, consideration is given to:

- The goals of the owner/finder/concerned bystander
- The needs of the animal
- The exigency of the situation
- The best possible solution given capacity and resources in the shelter and community

This assessment may take place over the phone, via a web-form, or in some cases even by simply using flow charts and criteria provided on a website. The resulting response may range from a recommendation for immediate intake (for instance, of an injured or dangerous animal), intake by appointment for non-urgent cases (e.g., healthy kittens old enough for adoption), or guidance to help manage the situation without shelter admission (e.g., resources to reunite found pets with their owners or strategies for co-existence and spay/neuter support for healthy community cats).

As more shelters replace *ad hoc*/unmanaged intake with appointment-based triage systems, the advantages of the latter have become increasingly apparent (8, 11). In addition to keeping more animals safe in the communities where they live, shelter workflow becomes more predictable, allowing more effective and efficient staffing. With fewer animals in the building, disease rates and associated medical costs tend to decrease (7, 14). Prioritizing the most vulnerable animals allows greater investment in each one. This may in part explain why, when COVID-19 related changes to U.S. shelters operations in 2020 resulted in a 22% decrease in intake, shelter euthanasia dropped in U.S. shelters by 49% (176,000 pets)—fewer animals in the system allowed a larger percentage to get the care needed to leave the shelter alive (9).

Reduced euthanasia and the ability to provide better care for animals in turn often results in greatly improved staff morale (14). This is significant as workplace stress is commonly identified amongst animal shelter workers and may contribute to a variety of mental health issues and even elevated risk for suicide (15–17). Inadequate staffing, the inability to provide an appropriate level of care and a lack of control over the work environment have been linked to an increased risk of moral distress and burnout in animal and human healthcare settings (18–20). Euthanasia of animals is often a specific and potent additional stressor for shelter staff (20). Conversely, appointment-based triage offers greater opportunities for control and predictability of the work environment, and allows more opportunities for appropriate care to be provided either within the shelter or via community-based solutions. This may explain why some shelter staff reported paradoxically high levels of work satisfaction even in the very challenging early days of the COVID-19 pandemic.

Lower intake, reduced euthanasia, decreased disease rates and higher staff moral have the potential to trigger a virtuous cycle, freeing resources for greater investment in safety net services that improve community health and decrease the overall need for animal impoundment, as exemplified in this quote from one director of a public shelter from a survey of California shelter leaders regarding the impact of changes made in response COVID-19:

“*When the pandemic hit and we were able to use that to put in place more stringent managed intake policies, we were able to see a true difference in our ability to meet the 5 Freedoms, truly care for the animals, reduce LOS (length of stay), and also reduce our Net County Cost. The decrease in cost allowed us the ability to push through our plan to place a clinic building on the shelter property, which will open up so many opportunities to serve the public without increasing intake.”* (California shelter survey performed by UC Davis Koret Shelter Medicine Program, April 2021).

## Discussion

The COVID-19 pandemic revealed the hidden costs of “business as usual” in many sectors of society. In animal shelters we now recognize the costs of unmanaged intake as the flip side of the benefits of appointment-based triage: animals needlessly impounded; fewer pets reunited with their families; increased crowding, stress, disease and death of shelter animals; higher expenses and lower staff morale. Even more troubling, the burden of these negative consequences falls disproportionately on marginalized community members and their pets (21). The status quo system has resulted in animals from less affluent and more vulnerable communities being admitted to shelters at a higher rate, and leaving shelters alive at a lower rate in comparison to more affluent areas (22–25). This disparity has been linked to a variety of socioeconomic factors including poverty, housing insecurity, and ethnocultural factors (24, 26, 27). When intake is unplanned and chaotic, there is limited opportunity to understand and remediate a problematic situation, e.g., by offering short term care for pets of people experiencing a housing crisis, help with medical, food or other urgent care needs, spay/neuter services or other support to reduce intake and stabilize the valued connection between people and pets (28).

There is no reason to continue to tolerate such harms and inequities in association with animal shelters. Whether funded by public dollars or private donors, these organizations are intended to serve the common good of animals and people, keeping communities safe and supporting the connection between pets and families. Shelter staff and volunteers deserve the opportunity to provide humane care for animals and responsive customer service without struggling under an unmanageable burden or working within a chronically overwhelmed system. These ends can best be served by replacing *ad hoc*, unscheduled intake with a thoughtful, appointment-based system that allows situational assessment and response for all non-emergency requests. The decision of whether and when to admit an animal can then be made based on the capacity of the shelter and the needs of the animal and people involved, and guidance provided for finders, owners and concerned bystanders when shelter admission of an animal is not recommended.

## Conclusion

The COVID-19 pandemic created a systemic disruption that, while undeniably tragic, revealed opportunities to replace long-established norms with more humane, equitable and effective alternatives (2). The *ad hoc*, unscheduled intake model of sheltering so prevalent in North America arose over a century ago, undoubtedly with good intentions to optimize animal care and customer service; however, the experiences of many shelters during the pandemic built on existing research to prove the advantages of a more thoughtful, scheduled approach. The success of triage and appointment-based care in human healthcare provides a roadmap for shelters to similarly match the type and timing of the response with the needs of those seeking care, the exigency of the situation and the capacity of the organization. In so doing, shelters lay the foundation for more equitable, humane and sustainable systems that will better serve animals and communities in the years to come.

## Data Availability Statement

The original contributions presented in the study are included in the article/supplementary material, further inquiries can be directed to the corresponding author.

## Author Contributions

The author confirms being the sole contributor of this work and has approved it for publication.

## Conflict of Interest

The author declares that the research was conducted in the absence of any commercial or financial relationships that could be construed as a potential conflict of interest.

## Publisher's Note

All claims expressed in this article are solely those of the authors and do not necessarily represent those of their affiliated organizations, or those of the publisher, the editors and the reviewers. Any product that may be evaluated in this article, or claim that may be made by its manufacturer, is not guaranteed or endorsed by the publisher.
